# A novel technique of using computer imaging for efficient detection of M. tuberculosis in acid-fasta stain procedure

**DOI:** 10.1186/1753-6561-5-S6-P30

**Published:** 2011-06-29

**Authors:** HC Huang, KL Kuo, CF Wei, MY Yen, YE Lin

**Affiliations:** 1 Taipei City Hospital, Taipei, China; 2 National Kaohsiung Normal University, Kaohsiung, Taiwan, China

## Introduction / objectives

Tuberculosis is an emerging infectious disease in Taiwan which nearly 15,000 newly diagnosed cases are reported each year. The most economical method for laboratory diagnosis of pulmonary tuberculosis is based on the bacteriological examination of sputum smears stained by the Acid- Fast stain method for acid fast bacilli (AFB). Thus, microscopic examination of specimens for AFB plays a key role in the initial diagnosis, monitoring of treatment, and eligibility for release from isolation. However, laboratory technicians often time are overwhelmed with large quantity of smears and under pressure of limited time allowed to issue the results. In this study, we propose a novel technique to transfer microscopic image of AFB from the eyepiece to a large size computer LCD screen via a digital camera embedded on the microscope.

## Methods

We compared the results between Kinyoun acid fast stain smears under computer screen and M. tuberculosis cultures. The microscopic images were digitally stored in computer and shown on the LCD monitor for analysis.

## Results

Total 494 sputum specimens were processed for AFB. Using culture as the gold standard, our results show that the accuracy, sensitivity and specificity of our method is 53.0% (262/494), 48.0% (183/375), and 66.3% (179/119), respectively. Although our method is low in accuracy and sensitivity, but our specificity is higher than other commonly used method. The time required for each slide also cut in half.

## Conclusion

Our proposed method may combine with computerized pattern recognition software to automatically detect AFB from images which would significantly increase the accuracy and sensitivity. Such digital image automation may be the future for promoting better laboratory quality and efficiency.

## Disclosure of interest

None declared.

